# Amino acids intake and physical fitness among adolescents

**DOI:** 10.1007/s00726-017-2393-6

**Published:** 2017-03-17

**Authors:** Luis Gracia-Marco, Silvia Bel-Serrat, Magdalena Cuenca-Garcia, Marcela Gonzalez-Gross, Raquel Pedrero-Chamizo, Yannis Manios, Ascensión Marcos, Denes Molnar, Kurt Widhalm, Angela Polito, Jeremy Vanhelst, Maria Hagströmer, Michael Sjöström, Anthony Kafatos, Stefaan de Henauw, Ángel Gutierrez, Manuel J. Castillo, Luis A. Moreno, On behalf of the HELENA Study Group

**Affiliations:** 1 CHERC (Children’s Health and Exercise Research Centre), College of Life and Environmental Sciences, Sport and Health Sciences, St. Luke’s Campus, University of Exeter, Heavitree Road, Exeter, EX1 2LU Devon UK; 2 GENUD “Growth, Exercise, NUtrition and Development” Research Group, University of Zaragoza, Saragossa, Spain; 3 School of Public Health, Physiotherapy and Sports Science, National Nutrition Surveillance Centre, University College Dublin, Dublin, Ireland; 4 Department of Medical Physiology, School of Medicine, University of Granada, Granada, Spain; 5 Department of Physical Education, Faculty of Education, Cádiz University, Cádiz, Spain; 6 ImFine Research Group, Department of Health and Human Performance, Faculty of Physical Activity and Sport Sciences (INEF), Universidad Politécnica de Madrid, Madrid, Spain; 7 CIBER: CB12/03/30038 Fisiopatología de la Obesidad y la Nutrición, CIBERobn, Instituto de Salud Carlos III (ISCIII), Madrid, Spain; 8 Department of Nutrition and Dietetics, Harokopio University, Athens, Greece; 9 Immunonutrition Group, Institute of Food Science and Technology and Nutrition, ICTAN-CSIC, Madrid, Spain; 10 Department of Paediatrics, University of Pécs, Pécs, Hungary; 11 Division of Clinical Nutrition, Department of Paediatrics and Adolescents Medicine, Medical University of Vienna, Vienna, Austria; 12 Department of Pediatrics, Private Medical University Salzburg, Salzburg, Austria; 13 Council for Agricultural Research and Economics, Research Centre for Food and Nutrition, Rome, Italy; 14 LIRIC, Lille Inflammation Research International Center, University of Lille, Inserm, CHU Lille, UMR995, 59000 Lille, France; 15 Centre d’investigation clinique, University of Lille, Inserm, CHU Lille, CIC 1403, 59000 Lille, France; 16 Department of Bioscience and Nutrition, Karolinska Institutet, Solna, Sweden; 17 Department of Social Medicine, Preventive Medicine and Nutrition Clinic, School of Medicine, University of Crete, Crete, Greece; 18 Department of Public Health, Ghent University, Ghent, Belgium; 19 Facultad de Ciencias de la Salud, Universidad de Zaragoza, Saragossa, Spain

**Keywords:** Diet, Cardiorespiratory fitness, Muscular fitness, Carbohydrates, Youth

## Abstract

The aim was to investigate whether there was an association between amino acid (AA) intake and physical fitness and if so, to assess whether this association was independent of carbohydrates intake. European adolescents (*n* = 1481, 12.5–17.5 years) were measured. Intake was assessed via two non-consecutive 24-h dietary recalls. Lower and upper limbs muscular fitness was assessed by standing long jump and handgrip strength tests, respectively. Cardiorespiratory fitness was assessed by the 20-m shuttle run test. Physical activity was objectively measured. Socioeconomic status was obtained via questionnaires. Lower limbs muscular fitness seems to be positively associated with tryptophan, histidine and methionine intake in boys, regardless of centre, age, socioeconomic status, physical activity and total energy intake (model 1). However, these associations disappeared once carbohydrates intake was controlled for (model 2). In girls, only proline intake seems to be positively associated with lower limbs muscular fitness (model 2) while cardiorespiratory fitness seems to be positively associated with leucine (model 1) and proline intake (models 1 and 2). None of the observed significant associations remained significant once multiple testing was controlled for. In conclusion, we failed to detect any associations between any of the evaluated AAs and physical fitness after taking into account the effect of multiple testing.

## Introduction

Physical fitness has been associated with health-related outcomes in children and adolescents (Ortega et al. 2008b). Results from longitudinal studies indicate that a higher level of physical fitness in young population is associated with a healthier cardiovascular profile when they become adults (Ruiz et al. 2009).

Although physical fitness is in part genetically determined, it is also influenced by environmental factors, mainly physical activity, and it is not well understood how it is associated with nutrition. Few studies have examined the association between dietary intake and physical fitness in adults; overall, they conclude that a healthy diet is positively associated with cardiorespiratory fitness (CRF) levels (Brodney et al. 2001; Haraldsdottir and Andersen 1994; Shikany et al. 2013). We previously observed a higher intake of dairy products and bread/cereals and a lower consumption of sweetened beverages in adolescents with high CRF (Cuenca-Garcia et al. 2012). However, specific macronutrients need still to be studied in detail due to their potential physiological interaction with physical fitness. Dietary protein and, more specifically, intakes of specific amino acids (AA) contribute to the growth, repair and maintenance of muscle cells and thus in the physical performance (Phillips 2012). In this line, dietary AA supplementation seems to be associated with muscle growth and athletic performance (Wu 2009). Branched-chain AA (BCAA), among others, help maintaining muscle tissue and are required when doing physical exercise (Matsumoto et al. 2009; Shimomura et al. 2004). However, no evidence exists yet about the specific role that AA might play on physical fitness. We hypothesize that higher intakes of AA might be associated with higher levels of muscular fitness and CRF, due to their physiological effects on muscle cells during and after exercise. In addition, dietary carbohydrates are one of the main energy sources for prolonged and low-intensity physical activities, and short, high-intensity exercises (Correia-Oliveira et al. 2013). Therefore, they have to be considered when examining the relationship between AA intake and physical fitness.

To our knowledge none has examined yet the association between the intake of a large number of AA and muscular fitness and CRF among adolescents. Since physical fitness has been associated with health outcomes, investigating whether dietary AA are positively related to muscular fitness and CRF is of both clinical and public health relevance. The purpose of this study is to investigate whether there is an association between AA intake and physical fitness in European adolescents and if so, to assess whether this association is independent of carbohydrates intake.

## Methods

The current study is based on data derived from the Healthy Lifestyle in Europe by Nutrition in Adolescence cross-sectional study (HELENA-CSS) in which 3528 boys and girls aged 12.5–17.5 years had valid data for gender and body mass index (BMI). A subsample of 1481 adolescents (51.6% girls) were included in this report based on the following inclusion criteria: valid data on gender, BMI, AA intake, muscular fitness, CRF, physical activity (PA) and two 24-hour dietary recalls (24-HDR). Adolescents from the entire HELENA cohort were significantly older, weighed more and had higher mean BMI (all p < 0.05) (data not shown) than those included in this study.

The study was approved by the Research Ethics Committees of each city involved and was performed following the ethical guidelines of the Declaration of Helsinki, 1964 (revision of Edinburgh 2000). A written informed consent form was obtained from the adolescents and their parents.

A physical examination was performed with participants barefoot and wearing underwear. Briefly, body weight was measured with an electronic scale (Type SECA 861; range 0.05–130 kg; precision 0.05 kg). Height was measured in the Frankfurt plane with a telescopic height measuring instrument (Type SECA 225; range 60–200 cm; precision 1 mm). BMI was calculated as body weight (kg) divided by the height squared (m^2^).

Physical fitness was measured using tests that have been shown to be reliable in young people (Ortega et al. 2008a). The handgrip test (kg) was used to assess upper limbs muscular fitness. The ratio between handgrip and body mass was used in this report due to their significant correlation (*r* = 0.67 and *r* = 0.48 in boys and girls, respectively; all *p* < 0.001). In addition, previous studies have observed that weight status plays a positive role in handgrip performance in adolescents (Artero et al. 2010). The standing long jump test (cm) was used to assess lower limbs muscular fitness. The ratio between standing long jump and height was used in this report. The 20-m shuttle run test (stage) was used to assess CRF and VO_2max_ (ml/kg/min) was estimated (Leger et al. 1988). As results from this study indicated that *V*O_2max_ (ml/kg/min) penalized heavier adolescents, *V*O_2max_ was expressed relative to body mass as a power function ratio standard (Tolfrey et al. 2006), with body mass raised to the power 0.77 (ml/kg^−0.77^/min). The suitability of an exponent of 0.77 was determined by log–log transformations and subsequent linear regression on the raw data.

Dietary intake was assessed by the HELENA-DIAT (Dietary Assessment Tool), a self-administered computer-based tool shown to accurately assess dietary information of European adolescents (Vereecken et al. 2008). Two non-consecutive 24-HDR within a time span of 2 weeks were obtained from each participant during school time and assisted by fieldworkers. The German Food Code and Nutrition Data Base (Bundeslebensmittelschlüssel, BLS Version II.3.1) (Dehne et al. 1999) was used to calculate energy and nutrient intakes. The usual food and nutrients intake was estimated by the Multiple Source Method which takes into account the within-person variability of the dietary data (Harttig et al. 2011). Energy intake was estimated in kilocalories per day (kcal/d), carbohydrate, protein and fat intake in grams per day (g/d) and grams per kilograms of body weight and per day (g/kg/d) and AA intake in milligrams per day (mg/d).

The Family Affluence Scale (FAS) is a valid socioeconomic status index in young people and has been previously used in large epidemiologic studies. It is based on the concept of material conditions in the family related to family expenditure and consumption (affluence) (Currie et al. 1997). The answers from all the questions were summed (range 0–8) and then grouped into three levels: low (0–2), medium (3–5), and high (6–8).

Uni-axial accelerometers (Actigraph MTI, model GT1M, Manufacturing Technology Inc., Fort Walton Beach, FL, USA) were used to objectively measure PA. At least three days of recording, with a minimum of 8 h registration per day, was set as an inclusion criterion. The time sampling interval (epoch) was set at 15 s. Average PA, expressed as mean counts per minute was used as a measure of overall PA.

Analyses were performed using the Statistical Package for Social Sciences software (SPSS, version 21.0 for WINDOWS; SPSS, Chicago, IL, USA) and values of *p* < 0.05 were considered statistically significant. After log-transformation of AA intakes, all variables showed a normal distribution. Since interactions between sex and the studied variables were observed (*p* < 0.05), results are given separately by sex. Descriptive data were assessed by one-way ANOVA for normally distributed variables and by Mann–Whitney *U* test for non-normally distributed variables. In case of categorical variables, the Chi-squared test was applied. Pearson correlation coefficients were calculated to analyse the association between total carbohydrate intake (g/day) and physical fitness. The association between AA intakes (independent variables) and fitness tests (dependent variables) was examined by multilevel linear regression analysis. Study centre was included as random intercept. Age, FAS, average PA and total daily energy intake (kcal/day) were entered as covariates in model 1. Model 2 included covariates from model 1 plus total carbohydrate intake (g/d). Significant associations (*p* < 0.05) found in the multilevel linear regression analyses (models 1 and/or 2) were examined more in depth by analyses of covariance (ANCOVA). Tertiles of AA intakes were entered as fixed factor, physical fitness variables were entered as dependent variables and study centre, age, FAS, average PA, total daily energy intake (kcal/day) and carbohydrate intake (g/day) were entered as covariates.

## Results

Descriptive data are provided in Table 1. Except for mean age and BMI all other analysed traits differed by gender.

**Table 1:** Descriptive characteristics of the population sample

	All (*n* = 1481)		Boys (*n* = 714)		Girls (*n* = 767)		*p*
Age (years)	14.7	1.2	14.8	1.3	14.7	1.2	0.194
Weight (kg)	56.7	11.4	59.4	12.7	54.2	9.3	<0.001
Height (cm)	165.4	9.1	169.5	9.5	161.9	7	<0.001
BMI (kg/m^2^)	20.6	3.2	20.5	3.3	20.6	3.1	0.433
*V*O_2max_ (ml/kg/min)	42.1	7.4	46.2	7.1	38.4	5.5	<0.001
Hand grip (kg)	30.9	8.8	35.9	9.3	26.2	4.9	<0.001
Hand grip/weight	0.5	0.1	0.6	0.1	0.5	0.1	<0.001
SLJ (cm)	167.2	34.4	187.8	30.5	148	25.5	<0.001
SLJ/height	1	0.2	1.1	0.2	0.9	0.2	<0.001
FAS (%)							
Low	10.8		8.1		13.2		
Medium	56.3		56.7		56.1		0.004
High	32.9		35.2		30.7		
Average PA (cpm)	405.2 (323.5–522)		472 (371.2–595.4)		359.6 (297.9–437.2)		<0.001

ANOVA was performed for normally distributed variables [mean (SD)] and Mann–Whitney *U* test for non-normally distributed variables (median (interquartile intervals)

Percentages were calculated for categorical variables and the Chi-squared test was applied

*BMI* body mass index, *CPM* counts per minute, *FAS* family affluence scale, *PA* physical activity, *SLJ* standing long jump, *VO*_*2max*_ maximal oxygen consumption

**Fig. 1:**
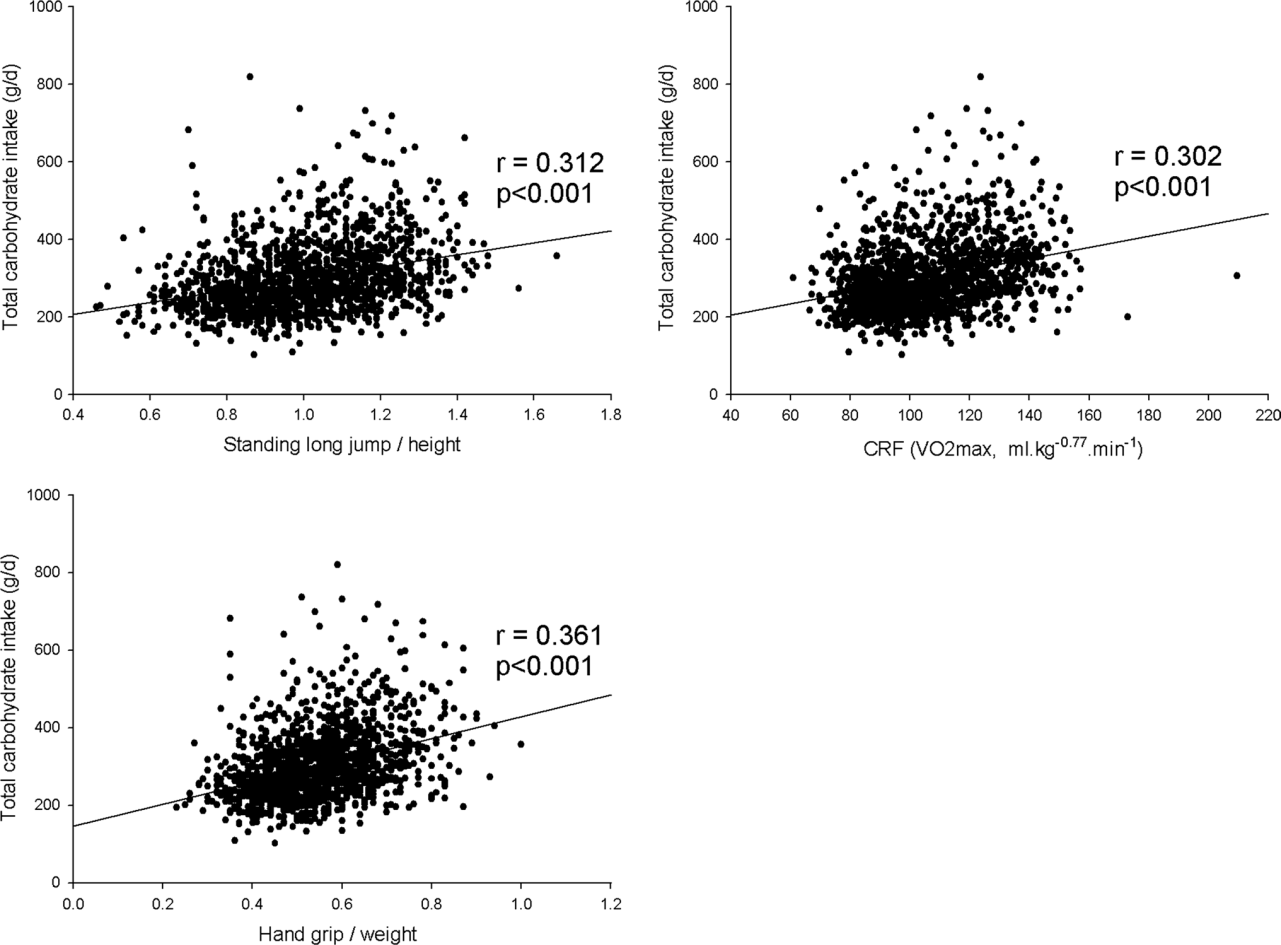
Pearson correlation coefficients among the studied fitness variables and total carbohydrate intake (g/day). All *p* < 0.001

Dietary characteristics of the participants are provided in Table 2. Mann–Whitney *U* test showed that except for total carbohydrate intake (% energy), total protein intake (% energy) and total fat intake (% energy) all other analysed traits differed by gender. In addition, total carbohydrate intake (g/day) was significantly correlated with the physical fitness variables included in this study (*r* ranged from 0.302 to 0.361; all *p* < 0.001; Fig. 1).

**Table 2:** Dietary characteristics of the studied participants by sex

	All (*n* = 1481)		Boys (*n* = 714)		Girls (*n* = 767)		p*
	Median	25th–75th percentile	Median	25th–75th percentile	Median	25th–75th percentile	
Energy intake (kcal/day)	2329.2	2011.3–2777.2	2681.2	2302.8–3124.9	2078.1	1850.5–2357.0	<0.001
Total carbohydrate intake (g/day)	273.7	227.7–342.5	313.9	255.2–384.3	248.6	205.6–294.9	<0.001
Total carbohydrate intake (% energy)	49.1	45.0–52.9	48.8	44.7–52.7	49.3	45.5–53.1	0.157
Total carbohydrate intake (g/kg/day)	5.2	4.2–6.4	5.6	4.5–6.9	4.8	3.9–5.8	<0.001
Total protein intake (g/day)	89.3	72.4–108.6	102.5	85.2–126.2	77.7	65.6–93.8	<0.001
Total protein intake (% energy)	15.6	13.8–17.6	15.6	13.8–17.7	15.5	13.8–17.4	0.314
Total protein intake (g/kg/day)	1.6	1.3–2.0	1.8	1.5–2.3	1.5	1.3–1.8	<0.001
Total fat intake (g/day)	86.5	72.2–106.2	99.7	82.5–120.2	78.4	66.8–92.2	<0.001
Total fat intake (% energy)	32.4	28.4–36.5	32.3	28.3–36.0	32.5	28.5–36.7	0.224
Total fat intake (g/kg/day)	1.5	1.2–2.0	1.7	1.3–2.1	1.4	1.1–1.8	<0.001
Amino acids intake (mg/day)							
Alanine	4105.2	3321.7–5071.9	4678.9	3928.0–5948.7	3599.7	2977.1–4400.4	<0.001
Glycine	3627.1	2977.8–4445.9	4210.2	3483.9–5104.4	3220.1	2697.4–3795.4	<0.001
Isoleucine	4288.4	3515.0–5156.5	4922.0	4149.8–5944.6	3736.8	3188.4–4435.7	<0.001
Leucine	6969.5	5718.1–8373.6	7979.6	6794.5–9651.6	6098.4	5201.8–7168.4	<0.001
Valine	4860.2	3985.0–5832.6	5561.6	4734.0–6727.9	4224.7	3614.3–4977.3	<0.001
Phenylalanine	3951.6	3270.9–4753.1	4545.9	3876.9–5455.0	3445.0	2993.7–4052.4	<0.001
Tryptophan	1017.8	842.5–1219.4	1174.0	991.4–1405.2	895.7	771.2–1052.8	<0.001
Tyrosine	3165.0	2595.8–3819.6	3646.4	3089.9–4402.0	2765.0	2363.7–3256.9	<0.001
Arginine	4852.5	3994.3–5899.1	5542.0	4676.6–6801.7	4268.8	3605.1–5104.0	<0.001
Histidine	2431.5	1971.8–2924.4	2797.7	2352.1–3416.3	2127.6	1793.8–2520.5	<0.001
Lysine	5970.1	4800.9–7304.4	6768.4	5689.5–8472.7	5190.2	4323.7–6270.5	<0.001
Aspartate and asparagine	7781.0	6348.1–9414.9	8871.1	7521.5–10,919.7	6770.7	5773.3–8124.0	<0.001
Glutamate plus glutamine	17,713.4	14,858.6–21,337.5	20,343.3	17,444.8–24,180.0	15,520.7	13,601.6–18,249.4	<0.001
Serine	4278.6	3549.5–5134.5	4943.0	4219.9–5942.9	3738.7	3237.7–4359.2	<0.001
Threonine	3547.2	2899.7–4254.3	4050.6	3435.2–4945.9	3102.5	2644.5–3686.5	<0.001
Cysteine	1189.1	985.9–1428.5	1361.0	1172.3–1613.5	1043.2	899.1–1211.3	<0.001
Methionine	1951.9	1580.4–2364.1	2238.6	1886.5–2715.9	1707.7	1447.4–2037.5	<0.001
Proline	6281.4	5259.9–7668.7	7233.1	6232.5–8584.8	5563.9	4783.4–6422.6	<0.001

* *p* value obtained by means of Mann–Whitney *U* test

Multilevel linear regression analyses of the associations between specific AA intakes and physical fitness are displayed in Tables 3, 4 and 5. In boys, tryptophan, histidine and methionine were the only to be (positively) associated with lower limbs muscular fitness (Table 3) in model 1. However, these associations disappeared after adjusting for total carbohydrates intake (g/day). In girls, proline was the only AA positively associated with lower limbs muscular fitness (model 2). Also in girls, leucine (model 1) and proline (models 1 and 2) were positively associated with CRF (Table 4) while no association was found between AA intakes and CRF in boys. No significant associations were found among any of the AA and upper limbs muscular fitness neither in boys nor in girls (Table 5). In addition, analyses were re-run by replacing the confounding variable total carbohydrate intake (g/day) by total carbohydrate intake (% of energy) and the results did not vary (data not shown). However, none of the observed associations were significant after controlling for multiple testing (0.05/number of tests = 0.05/18 = 0.003).

**Table 3:** Mixed linear regression analysis addressing the association between amino acids (AA) intake and lower limbs muscular fitness in European adolescent boys and girls (*p* value set at 0.05)

AA intake (mg/day)*	Standing long jump/height							
	Boys (*n* = 714)				Girls (*n* = 767)			
	Model 1^a^		Model 2^b^		Model 1^a^		Model 2^b^	
	*β*	95% CI	*β*	95% CI	*β*	95% CI	*β*	95% CI
Aliphatic side chains								
Alanine	0.06	−0.01; 0.12	0.05	−0.02; 0.12	−0.01	−0.07; 0.05	0.00	−0.06; 0.07
Glycine	0.06	−0.01; 0.12	0.06	−0.01; 0.13	−0.02	−0.08; 0.04	−0.00	−0.07; 0.06
Isoleucine	0.06	−0.01; 0.14	0.06	−0.01; 0.14	0.02	−0.04; 0.09	0.04	−0.03; 0.12
Leucine	0.07	−0.01; 0.14	0.07	−0.01; 0.15	0.03	−0.04; 0.1	0.05	−0.02; 0.13
Valine	0.07	−0.01; 0.15	0.07	−0.02; 0.15	0.02	−0.05; 0.09	0.05	−0.03; 0.13
Aromatic side chains								
Phenylalanine	0.08	−0.01; 0.16	0.07	−0.01; 0.16	0.02	−0.05; 0.1	0.05	−0.03; 0.13
Tryptophan	**0.08**	**0.00; 0.16**	0.08	−0.01; 0.17	0.03	−0.04; 0.1	0.06	−0.02; 0.14
Tyrosine	0.07	−0.01; 0.14	0.07	−0.01; 0.15	0.03	−0.04; 0.09	0.05	−0.02; 0.13
Basic side chains								
Arginine	0.06	−0.01; 0.12	0.06	−0.01; 0.13	−0.02	−0.08; 0.04	−0.00	−0.07; 0.06
Histidine	**0.07**	**0.01; 0.13**	0.07	−0.01; 0.14	0.02	−0.05; 0.08	0.04	−0.03; 0.11
Lysine	0.05	−0.01; 0.11	0.05	−0.01; 0.12	0.01	−0.05; 0.05	0.02	−0.04; 0.08
Acidic side chains								
Aspartate and asparagine	0.05	−0.01; 0.12	0.05	−0.02; 0.12	−0.02	−0.08; 0.05	−0.00	−0.07; 0.07
Glutamate plus glutamine	0.08	−0.01; 0.17	0.08	−0.01; 0.17	0.04	−0.03; 0.12	0.06	−0.02; 0.14
Hydroxyl side chains								
Serine	0.07	−0.01; 0.15	0.06	−0.03; 0.16	0.02	−0.06; 0.1	0.04	−0.04; 0.12
Threonine	0.07	−0.01; 0.14	0.07	−0.01; 0.15	0.01	−0.05; 0.07	0.03	−0.04; 0.1
Sulphur-containing side chains								
Cysteine	0.07	−0.01; 0.16	0.07	−0.01; 0.16	−0.01	−0.08; 0.07	0.00	−0.08; 0.08
Methionine	**0.07**	**0.01; 0.13**	0.07	−0.01; 0.14	0.01	−0.05; 0.07	0.03	−0.04; 0.1
Cyclic side chain								
Proline	0.07	−0.02; 0.16	0.06	−0.02; 0.15	0.07	−0.01; 0.15	**0.08**	**0.00; 0.16**

Significant associations in bold (*p* < 0.05)

*AA* amino acids (log-transformed data), *CI* confidence intervals

* No significant associations were found once statistical significance was controlled for multiple testing (*p* < 0.003)

^a^ Model 1: adjusted by centre, age, family affluence scale, physical activity and total energy intake

^b^ Model 2: adjusted by model plus total carbohydrates intake (g/d)

**Table 4:** Mixed linear regression analysis addressing the association between amino acids (AA) intake and CRF in European adolescent boys and girls (*p* value set at 0.05)

AA intake (mg/day)*	CRF (*V*O_2max_, ml/kg^−0.77^/min)							
	Boys (*n* = 714)				Girls (*n* = 767)			
	Model 1^a^		Model 2^b^		Model 1^a^		Model 2^b^	
	*β*	95% CI	*β*	95% CI	*β*	95% CI	*β*	95% CI
Aliphatic side chains								
Alanine	0.65	−6.49; 7.78	−0.16	−8.13; 7.81	1.64	−3.64; 6.92	1.19	−4.65; 7.04
Glycine	1.8	−5.43; 9.04	1.27	−6.76; 9.31	0.99	−4.43; 6.41	0.42	−5.5; 6.34
Isoleucine	3.71	−4.26; 11.68	3.58	−5.47; 12.64	5.2	−0.61; 11.01	5.59	−0.94; 12.13
Leucine	3.83	−4.46; 12.13	3.66	−5.65; 12.97	**6.11**	**0.13; 12.09**	6.63	−0.04; 13.31
Valine	3.68	−4.9; 12.26	3.45	−6.28; 13.18	5.38	−0.78; 11.54	5.71	−1.18; 12.6
Aromatic side chains								
Phenylalanine	4.64	−4.46; 13.74	4.52	−5.58; 14.61	5.84	−0.67; 12.35	6.07	−1.07; 13.22
Tryptophan	5.25	−3.43; 13.91	5.45	−4.39; 15.29	5.89	−0.44; 12.21	6.34	−0.76; 13.44
Tyrosine	4.09	−4.13; 12.32	4.01	−5.35; 13.52	5.61	−0.24; 11.46	6.17	−0.47; 12.81
Basic side chains								
Arginine	1.34	−5.98; 8.66	0.68	−7.46; 8.84	0.58	−4.88; 6.03	−0.14	−6.18; 5.91
Histidine	2.57	−4.88; 10.02	2.19	−6.26; 10.65	3.24	−2.23; 8.71	3.16	−2.98; 9.29
Lysine	1.62	−4.87; 8.11	1.09	−6.42; 8.61	2.82	−1.91; 7.56	2.85	−2.65; 8.35
Acidic side chains								
Aspartate and asparagine	0.51	−6.99; 8.02	−0.32	−5.59; 7.96	0.12	−5.43; 5.67	−0.72	−6.87; 5.43
Glutamate plus glutamine	6.8	−2.85; 16.44	6.74	−3.47; 16.95	6.91	−0.11; 13.95	6.89	−0.45; 14.23
Hydroxyl side chains								
Serine	4.49	−5.06; 14.05	4.27	−6.31; 14.85	5.93	−0.8; 12.65	6.1	−1.23; 13.44
Threonine	2.62	−5.13; 10.37	2.21	−6.69; 11.12	4.06	−1.55; 9.67	4.23	−2.15; 10.62
Sulphur-containing side chains								
Cysteine	2.43	−6.75; 11.6	1.91	−7.77; 11.59	1.29	−5.52; 8.11	0.82	−6.25; 7.88
Methionine	2.89	−4.44; 10.22	2.66	−5.82; 11.14	3.74	−1.61; 9.09	3.88	−2.23; 9.99
Cyclic side chain								
Proline	7.5	−2.13; 17.14	7.38	−2.59; 17.36	**8.75**	**1.86; 15.64**	**8.73**	**1.66; 15.8**

Significant associations in bold (*p* < 0.05)

*AA* amino acids (log-transformed data), *CRF* cardiorespiratory fitness, *CI* confidence intervals

* No significant associations were found once statistical significance was controlled for multiple testing (*p* < 0.003)

^a^ Model 1: adjusted by centre, age, family affluence scale, physical activity and total energy intake

^b^ Model 2: adjusted by model 1 plus total carbohydrates intake (g/d)

**Table 5:** Mixed linear regression analysis addressing the association between amino acids (AA) intake and upper limbs muscular fitness in European adolescent boys and girls

AA intake (mg/d)*	Hand grip/weight								
	Boys (*n* = 714)					Girls (*n* = 767)			
	Model 1^a^			Model 2^b^		Model 1^a^		Model 2^b^	
	*β*		95% CI	*β*	95% CI	*β*	95% CI	*β*	95% CI
Aliphatic side chains									
Alanine	0.02		−0.02; 0.06	0.02	−0.02; 0.07	−0.01	−0.03; 0.03	0.01	−0.02; 0.04
Glycine	0.02		−0.02; 0.07	0.02	−0.02; 0.07	−0.01	−0.04; 0.02	−0.00	−0.04; 0.03
Isoleucine	0.03		−0.02; 0.07	0.03	−0.02; 0.08	0.01	−0.03; 0.04	0.02	−0.02; 0.06
Leucine	0.03		−0.02; 0.08	0.03	−0.03; 0.08	0.01	−0.03; 0.04	0.02	−0.02; 0.06
Valine	0.03		−0.02; 0.08	0.03	−0.02; 0.09	0.01	−0.03; 0.05	0.03	−0.01; 0.07
Aromatic side chains									
Phenylalanine	0.03		−0.02; 0.08	0.03	−0.03; 0.09	0.01	−0.03; 0.04	0.02	−0.02; 0.06
Tryptophan	0.03		−0.02; 0.08	0.03	−0.03; 0.09	0.01	−0.03; 0.05	0.03	−0.02; 0.07
Tyrosine	0.03		−0.02; 0.08	0.03	−0.02; 0.08	0.01	−0.02; 0.05	0.03	−0.01; 0.07
Basic side chains									
Arginine	0.02		−0.02; 0.07	0.02	−0.02; 0.07	−0.01	−0.04; 0.03	0.01	−0.03; 0.04
Histidine	0.03		−0.01; 0.07	0.03	−0.02; 0.08	0.01	−0.03; 0.03	0.02	−0.02; 0.05
Lysine	0.02		−0.02; 0.06	0.02	−0.02; 0.06	0.01	−0.02; 0.03	0.02	−0.01; 0.05
Acidic side chains									
Aspartate and asparagine	0.02		−0.02; 0.06	0.02	−0.03; 0.07	−0.01	−0.04; 0.03	0.01	−0.03; 0.04
Glutamate plus glutamine	0.04		−0.02; 0.09	0.03	−0.02; 0.09	0.00	−0.04; 0.04	0.01	−0.04; 0.05
Hydroxyl side chains									
Serine	0.03		−0.02; 0.09	0.03	−0.03; 0.09	0.01	−0.03; 0.05	0.02	−0.02; 0.07
Threonine	0.03		−0.01; 0.07	0.03	−0.02; 0.08	0.01	−0.03; 0.04	0.02	−0.02; 0.06
Sulphur-containing side chains									
Cysteine	0.03		−0.02; 0.08	0.03	−0.03; 0.08	−0.02	−0.06; 0.02	−0.01	−0.05; 0.03
Methionine	0.03		−0.01; 0.07	0.03	−0.02; 0.08	0.01	−0.03; 0.04	0.02	−0.02; 0.06
Cyclic side chain									
Proline	0.03		−0.03; 0.08	0.02	−0.03; 0.08	0.01	−0.03; 0.05	0.02	−0.03; 0.06

*AA* amino acids (log-transformed data), *CI* confidence intervals

*No significant associations were found (*p* > 0.05)

^a^ Model 1: adjusted by centre, age, family affluence scale, physical activity and total energy intake

^b^ Model 2: adjusted by model 1 plus total carbohydrates intake (g/d)

**Fig. 2:**
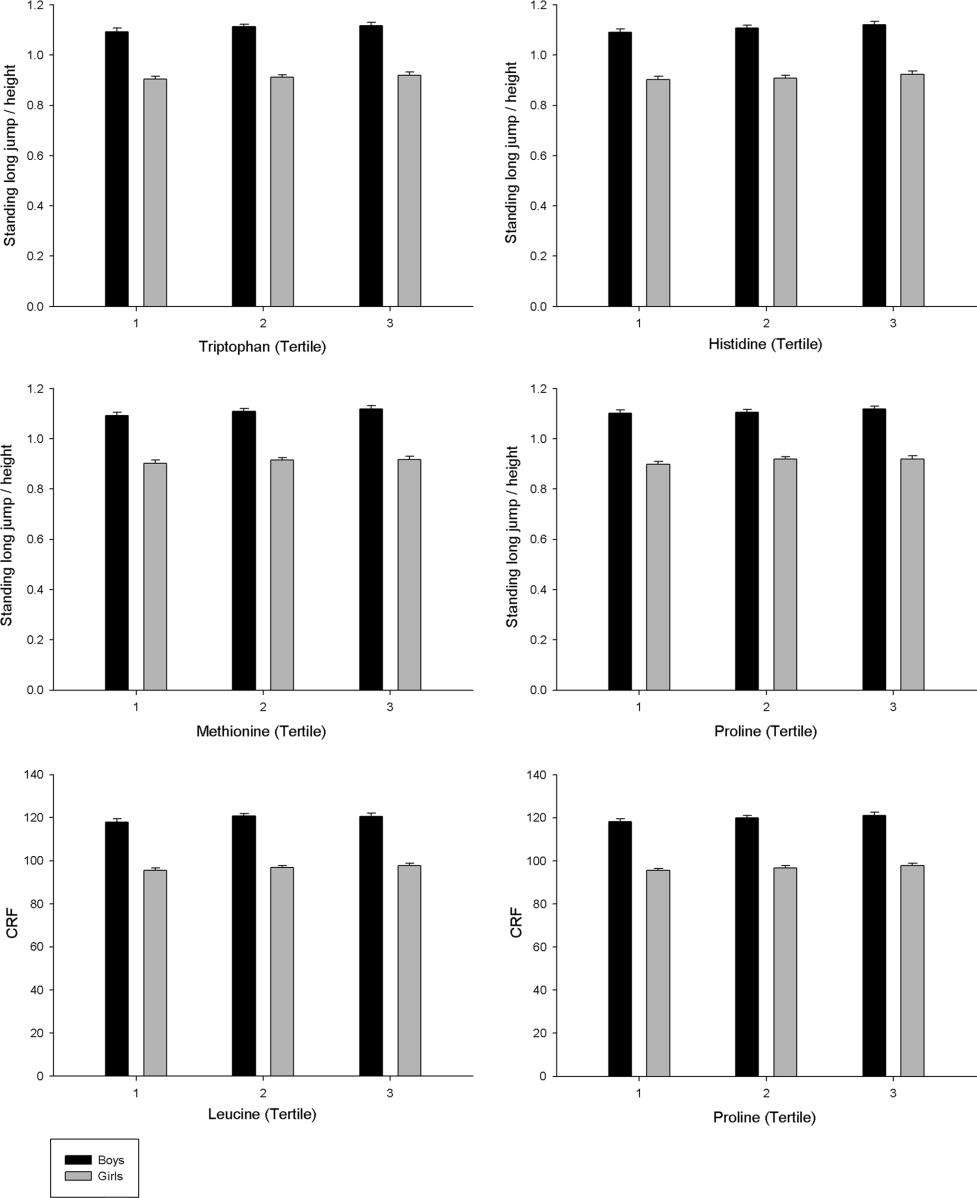
Differences in fitness according to AA intake (tertiles) in adolescents adjusted for study centre, age, family affluence scale, average physical activity, total daily energy intake and carbohydrate intake (g/day). Boys’ tertiles in mg/day: tryptophan (<1052.2, 1052.2–1289.8, >1289.8); histidine (<2483.5, 2483.5–3113.7, >3113.7); methionine (<1989.5, 1989.5–2496.1, >2496.1); leucine (<7154.7, 7154.7–8835.8, >8835.8); proline (<6538, 6538–8098.7, >8098.7). Girls’ tertiles in mg/day: tryptophan (<804.9, 804.9–987.6, >987.6); histidine (<1898.4; 1898.4–2367.5, >2367.5); methionine (<1509.6, 1509.6–1898.4, >1898.4); leucine (<5435.1, 5435.1–6766.1.8, >6766.1); proline (<5000.3, 5000.3–6063.6, >6063.6). Tertiles were calculated with raw data to be more meaningful although differences were examined with the log-transformed variables. All *p* > 0.05. *CRF* cardiorespiratory fitness

ANCOVA analyses of the associations between AA intake and physical fitness are shown in Fig. 2. Results showed that there were no significant differences between tertiles of AA intake and physical fitness neither in boys and girls.

## Discussion

To the best of our knowledge, this is the first study analysing the relationship between a large number of dietary AA and physical fitness in adolescents. AA intake was measured by means of two self-administered, computer-assisted, non-consecutive 24-HDR which have been shown to be appropriate in collecting detailed dietary data in adolescents (Vereecken et al. 2008). The physical fitness tests included in this study have been shown to be reliable in young people (Ortega et al. 2008a). By definition, essential AA (EAA) cannot be synthesized de novo by the organism and, therefore, they must be supplied in the diet. Pancreatic enzymes convert the diet-ingested proteins into AA in the lumen of the small intestine (Pasini et al. 2004). AA are absorbed from the small intestine and enter the portal vein for protein synthesis in skeletal muscle and other tissues (Wu 2016). Skeletal muscle has an active role in AA metabolism by synthesizing alanine and glutamine from circulating BCAA (Wu 2009). Furthermore, the skeletal muscle plays a key role during exercise and stores the biggest amount of AA in the body. It regulates the movement of AA (incorporate or release AA) according to the needs of the organism and the balance between catabolic and anabolic state. For example, when catabolism is prevalent (e.g. during exercise), AA are released by the skeletal muscle to subsequently be converted into glucose by the liver to help in the functioning of the glucose-dependent organs (Carubelli et al. 2015).

In our study, lower limbs muscular strength seems to be positively associated with some EAA such as tryptophan, histidine and methionine after controlling for centre, age, FAS, PA and total energy intake in boys. It is well known that an increase in muscle mass can be achieved via nutritional supplementation. Indeed, it has been suggested that dietary supplementation with one or a mixture of functional AA, such as leucine, proline and tryptophan, among others, may be beneficial for optimizing efficiency of metabolic transformations to enhance muscle growth and athletic performance (Wu 2009). However, increases in muscle mass do not always accompany increases in muscle strength. Previous studies in older women observed that EAA supplementation increased muscle mass but not muscle strength (Dillon et al. 2009; Kim et al. 2012). Interestingly, muscle strength only improved when exercise and AA supplementation were combined (Kim et al. 2012). In the current study with adolescents, PA was controlled for and significant associations only disappeared after adjusting for total carbohydrate intake, suggesting that a specific macronutrient such as carbohydrate has a stronger confounding role than the one exerted by PA or total energy intake in these associations. All significant reported associations were weak and disappeared after controlling for multiple testing.

In our study, CRF (*V*O_2max_) seems to be positively associated with an EAA such as leucine and a BCAA such as proline after controlling for centre, age, FAS, PA and total energy intake in girls. Once carbohydrate intake was considered as a covariate, the association between leucine and *V*O_2max_ disappeared. BCAAs account for 35% of the EAA in muscle proteins and 40% of the preformed amino acids required by mammals (Shimomura et al. 2004). BCAA help maintaining muscle tissue and are required during times of physical stress and intense exercise, characteristic of a *V*O_2max_ test. BCAA ingestion immediately before an incremental load exercise test following chronic (6-d) BCAA supplementation significantly increased *V*O_2max_ (Matsumoto et al. 2009) in young adults. BCAA excretion (leucine, isoleucine, and valine) was significantly lower in healthy adults with high fitness, as indicated by lower urinary levels of AA (Morris et al. 2013). As a response to exercise, AA biosynthesis and protein breakdown in skeletal muscle may increase (Rennie and Tipton 2000), which could possibly increase the systemic pool of AA. Therefore, exercise increases energy expenditure and as a consequence promotes oxidation of BCAA (Shimomura et al. 2004). Previous studies have also shown that EAA supplementation improves CRF in ambulatory chronic heart failure patients (Aquilani et al. 2008; Scognamiglio et al. 2008), with this being explained by improved muscle aerobic metabolism, prevalence of muscle anabolic processes and reduction of insulin resistance. Our results from a sample of healthy adolescents suggest that AA intake may have a positive influence on physical fitness because of the AA’s removal by active skeletal muscle during exercise and the increase in oxidation as exercise progresses. However, these findings should be interpreted cautiously as observed associations were weak and might be simply due to chance. In fact, no associations are found once statistical significance is controlled for multiple testing.

Model 2 was adjusted for carbohydrates intake to account for any potential confounding role that it might play in the association between AA and physical fitness. Dietary carbohydrates are one of the main fuels for sport activities, and their relevance for optimal sport performance is undisputed among experts, improving performance in both prolonged, low-intensity and short, high-intensity exercises (Correia-Oliveira et al. 2013). In general, there is a consensus claiming an ergogenic effect of carbohydrates ingested just before or during a performance bout (Colombani et al. 2013). Carbohydrate feeding prior to exercise provides additional supplies for oxidation, resulting in increased muscle glucose uptake and reduced liver glucose output during exercise, and enhanced blood glucose availability which may preserve muscle glycogen stores (Jamurtas et al. 2011). In addition, higher carbohydrates intake is accompanied of higher insulin secretion, which is a determining factor of AA incorporation into muscle cells and proteins (Gower and Goss 2015). The fact that significant associations between AA intake and lower limbs muscular strength and CRF disappeared after controlling for carbohydrates intake could reflect that those adolescents that performed better in both physical fitness tests might have had higher carbohydrates intake compared to those who did worse. It is likely that these adolescents had also higher daily PA levels, explaining their higher carbohydrates consumption, as main energy source, which may occur along with an increased intake of proteins, as AA precursors, to enhance muscle development.

Despite the lack of significant associations in this sample of European healthy adolescents, it is noteworthy to highlight the key role of protein nutrition on health. Adolescence is a period of rapid development which entails increased tissue generation and protein gain; therefore, protein requirements are increased and adequate protein intake is crucial for optimal growth and, in the long term, for healthy ageing (Wu 2016). Although previous research has shown a decrease in PA levels in adolescents (Ruiz et al. 2011), mainly among girls, adolescence is still characterized for high levels of PA performance as compared to other periods of life. While sedentary behaviour seems to exert a detrimental effect on skeletal muscle, dietary protein and moderate exercise have synergistic effects on the protein synthesis of skeletal muscle (Wu 2016). Furthermore, evidence shows that PA combined with an increased intake of high-quality proteins may represent an effective strategy to enhance fat loss while preserving muscle mass (Wu 2016). Protein is a major component of bones and plays a key role in skeletal health to reduce risk for osteopenia and osteoporosis by regulating the efficiency of the absorption of dietary minerals and bone mineralization; high protein intake, however, can contribute to bone loss with the stimulation of calcium urinary excretion (Bonjour 2011). In this regard, dietary protein intake has also been linked to negative health outcomes. Excessive protein intake may cause intestinal, hepatic, renal and/or cardiovascular dysfunction in healthy people (Pedersen et al. 2013) and large animal protein intakes could be associated with an increase in risks of cancer and diabetes (Levine et al. 2014; van Nielen et al. 2015).

The cross-sectional design of this study does not allow for causality interpretations. Increasing the number of recording days would have been desirable to compensate for day-to-day variability in the 24HDR; however, dietary data were corrected for between- and within-person variability to partially mitigate this limitation and adolescents’ usual intakes were calculated using the Multiple Source Method to obtain more accurate intake estimates (Harttig et al. 2011). The most exhaustive food composition table available in Europe was used to compute nutrient intakes (Dehne et al. 1999). Although variability in nutrient content across countries is always present, the applied food composition table was considered a good alternative to national food composition tables (Julian-Almarcegui et al. 2016). Nevertheless, dietary assessment methods are subject to measurement error and it cannot be precluded certain degree of inaccuracy when computing nutrient intakes, including amino acids intake. Despite the aforementioned limitations, this is the first study reporting the association between different physical fitness components and a large number of AA in adolescents. The fitness tests used in the present report have shown a good criterion-related validity in adolescents. Bonferroni correction was applied to counteract for the multiple testing problem, which is considered the most conservative method to control the familywise error rate.

## Conclusions

We failed to detect any associations in this sample of healthy European adolescents between any of the evaluated AAs and physical fitness after taking into account the effect of multiple testing.
